# Association between HSV1 Seropositivity and Obesity: Data from the National Health and Nutritional Examination Survey, 2007–2008

**DOI:** 10.1371/journal.pone.0019092

**Published:** 2011-05-11

**Authors:** Zuzana Karjala, Diane Neal, James Rohrer

**Affiliations:** 1 Walden University, Minneapolis, Minnesota, United States of America; 2 Mayo Clinic, Rochester, Minnesota, United States of America; University of Nebraska – Lincoln, United States of America

## Abstract

**Background:**

Herpes simplex virus (HSV) is among the most common sexually transmitted pathogens in the United States and worldwide. HSV has a high incidence of undetected cases. In addition, there is no treatment, and there is a lack of knowledge why disparities among populations exist. Research studies suggest that fat tissue may participate in body's immune responses, and the impact of obesity on susceptibility to HSV1 infection is not clear. The purpose of this study was to examine whether obesity is a risk factor for HSV1 infection using a large sample from the general population.

**Methods/Principal Findings:**

This cross-sectional study used data from the National Health and Examination and Nutritional Examination Survey (NHANES) from 2007–2008. Variables, gender, age, race/ethnicity, marital status, education, poverty level, and diabetes represented potential confounders and were included in analyses. The two-tailed Pearson's chi square, student's t test, and a multiple logistic regression analysis were applied to evaluate associations using a significance value of p≤0.05. Adjusted odds ratios with 95% confidence interval represented the degree of these associations. The prevalence of HSV1 infection in US population between 20 and 49 years old was 60.3% (n = 1,536). In this study, having a BMI classified as the obese group (BMI 30–39.9) was significantly associated with HSV1 infection before [unadjusted OR = 1.74 (95% CI 1.20–2.51), p = 0.006] and after controlling for socio-demographic factors [adjusted OR = 1.50 (95%CI 1.06–2.13)], p = 0.026]. This association was stronger than three already established risk factors of age, female gender, and poverty level.

**Conclusions/Significance:**

This study provides evidence that obesity may play a role in the susceptibility to HSV1 infection. Findings from this study suggest that obesity should be considered when designing preventive measures for HSV1 infection. These results may also explain why some people acquire HSV1 infections and some do not. Further, these findings may justify an increased emphasis on the control and prevention of HSV1 transmission and other pathogens in overweight and obese populations.

## Introduction

HSV is an intracellular pathogen that can affect the skin of multiple parts of the body including anogenital region, mouth, eyes, nervous system, and various major organs [1], [2], [3]. HSV is contagious and humans are the only known reservoir [4].

HSV belongs to a family of Herpesviridae, subfamily Alphaherpesvirinae. HSV is a fairly large, enveloped virus containing a double stranded DNA encoding for more than 80 proteins, including enzymes directly involved in binding to cells, fusion, synthesis, and replication. HSV usually infects epithelial mucosal cells, in which viruses replicate. The replicated virus follows nerve cells and usually settles in the trigeminal ganglia or sacral ganglia where it can become dormant. The dormant virus can be reactivated at any time by various stressors including sun, fever, or psychological stress [4]. Reactivated virus can travel back to the original point of entry and repeat the cycle.

HSV triggers cell mediated immune response, which peaks between 7 to 14 days after infection, then declines and reaches constant levels after 1 or 2 months [5], [6]. The symptoms may be mild and not obvious to the carrier and for this reason, HSV infections may often be unrecognized. It has been documented that HSV infection may allow entry of other pathogens (e.g., Human Immunodeficiency Virus), and the infected person may also be at higher risk for diseases including bacterial vaginosis, oropharyngeal cancer, cardiovascular diseases (CVD), and metabolic disorders [4], [7], [8], [9]. HSV has the ability to “trick” the body's immune system by binding Immunoglobulin Gamma (IgG), which enables the virus to go unnoticed, leading to persistent and recurrent infections [10]. As it is extremely difficult to rid the body of this infection, the condition is a chronic one.

HSV is usually transmitted via direct contact with infected discharge from blisters, and the primary infection may occur at any stage of life. HSV are commonly divided into two primary subtypes, HSV1 and HSV2, and these subtypes can be distinguished antigenically [10]. Although both types can cause infections around the mouth or the genital region, it is estimated that more than 90% of oral infections (e.g., lips, mouth, tongue, or pharynx) are caused by HSV1, and HSV2 is usually transmitted through sexual contact [4], [11]. Recently, and in contrast to this established pattern, public health authorities have started noticing a shift in the prevalence of genital herpes caused by HSV1 in some populations [12], [13], and certain geographic locations [12], [14]. It has been hypothesized that this shift could be a result of a change in sexual practices (e.g., more common oral sex) and/or increased incidence of using condoms to prevent HSV2 transmission [13]; however, further research will be needed to confirm these theories.

The shift in HSV ratio may have serious consequences. For example, mothers infected during pregnancy or delivery can expose their newborn babies to the risk for developing neonatal herpes infection, which often leads to neurologic disorders or death [15], [16]. Findings from one epidemiological study on the prevalence of HSV in Canada indicated that more than 60% of newborns with HSV were caused by HSV1. This finding suggests that without effective preventive programs we may see an increase in this type of transmission. The transmission may occur prenatally, perinatally and after the birth; therefore, the emphasis on prevention may be especially important in young women and mothers [17]. A recently noted change in the HSV1/HSV2 ratio (i.e., increase in HSV1 and decrease in HSV2) in some populations suggests that new epidemics could be evolving [13]. There is no effective treatment for HSV infection, and preventive measures currently used are inefficient [18]. The identification of all risk factors involved in HSV1 prevalence is essential to alleviate the burden of HSV infection.

Epidemiological studies indicate that obese and overweight people are at higher risk for postoperative infections and mortality associated with infections [19], [20]. The increased seropositivity for specific pathogens has been identified in obese individuals [21], [22]. For example, the association of adenovirus-36 with obesity in humans and animals has been well documented [23], [24]. Other infectious agents including *Chlamydia pneumoniae* (*Cpn*) and *Helicobacter pylori* (*Hp*) have been also suggested to be associated with obesity [25], [26]. However, the association with other pathogens, such as HSV1, still needs to be evaluated. The aim of this study was to determine whether obesity is a potential risk factor for HSV1 infection.

## Methods

This cross-sectional study used NHANES data from 2007–2008. NHANES data were collected by the National Center for Health Statistics (NCHS) and are publicly accessible at http://www.cdc.gov/nchs/nhanes/nhanes2007-2008/nhanes07_08.htm [27]. A total of 12,943 participants were included in the NHANES 2007–2008. Among those participants, 3,364 individuals were tested for HSV1 antibodies. After excluding 3 participants from HSV1 testing, 3,361 participants for HSV1 had a known HSV1 serostatus. Only participants between 20 and 49 years of age who had complete records for all tested variables were included in the analyses and the final sample size used for analyses was 2,546.

In this study, HSV1 infection represented by seroprevalence (positive, negative) was a dependent variable, and body mass index representing the degree of obesity status (BMI<25 for normal weight, BMI = 25–29 for overweight, BMI 30–39.9 for obese and BMI>40 for severely obese). Age, gender, race/ethnicity marital status, poverty level, and diabetes were used as covariates. The differences between groups for the continuous variable, age, was tested by student's *t* test. All other variables were categorized, and the differences between groups were tested by Pearson's chi-square test. Multivariate logistic regression was used to examine the association between the dependent variable, HSV1 infection, and independent variable before and after the adjustment for possible confounding factors. Odds ratios with 95% confidence interval were used to represent associations and p values less than 0.05 was considered statistically significant.

### Determination of HSV infection

The dependent variables for this study were seropositivity with HSV1. To determine serostatus, eligible study subjects provided a blood sample at the mobile examination center. Blood serum was sent for further processing to Emory University, where laboratory experts used enzymatic immunodot assay (EIA) to detect specific antibodies.

### Determination of Obesity status

The independent variable for this study was obesity status representated by Body mass index (BMI). During NHANES data collection, the body weight and height measurements were performed by trained technician using calibrated equipment. The body mass index was calculated by dividing weight (kg) by height (m^2^). In this study, BMI was divided into four categories; BMI<25 was considered normal weight, BMI = 25–29 was considered overweight, BMI 30–39.9 was considered obese and BMI>40 was considered severely obese.

### Determination of Covariates

Other variables were obtained from NHANES questionnaire and, except for age, were also divided into categories. In all analyses, age was treated as a continuous variable. All respondents over age of 20 were asked “Other than during pregnancy, have you ever been told by a doctor or other health professional that you have diabetes or sugar diabetes?”. For the purposes of this study, the participants who answered yes or bordeline were considered diabetic; participants who answered no were considered nondiabetic. The highest educational level of participants was based on the response to:“What is the highest grade or level of school you have completed or the highest degree you have received?”. To be consistent with associated literature, the education levels were divided into 3 categories “Less than high school”, “High school graduate/GED” and “Higher”.

Gender was assigned as “Male” and “Female”. In this study, the marital status was divided into two categories; single and married. The category “single” included widowed, divorced, separated, never married responses and “married” included married and living with a partner responses.

The race/ethnicity category was determined from the question asked by the interviewer “What race do you consider yourself to be?”. The categories used in this study were as follows; Non Hispanic White, Non Hispanic Black, Mexican American, and Other race/ethnicities.

The poverty level was based on poverty income ratio (PIR) included in the questionnaire. PIR was determined based on the most accurate estimate for family income per year and family size. In this study, the poverty level was categorized as follows; PIR<1 was labeled as poor and PIR≥1 was considered as not poor.

### Statistical Analysis

All data management and analyses were performed using STATA 11.0 software [28]. The downloaded data were checked for integrity before and after merging. NHANES uses a complex study design that requires the use of weighted methodology and proper clustering during the analytical process [27]. To address issues of unequal probabilities of selection and modification for missing responses, appropriate weight variables (i.e., wtmec2yr, sdmvstra, and sdmvpsu) were used in all statistical analyses. These weight variables were generated by NHANES in order the sample to represent the U.S. civilian non institutionalized Census population [27].

Bivariate analyses were performed to evaluate descriptive characteristics of the study participants and to evaluate dependent and independent variables. The bivariate analyses were performed for the association of HSV1 and the independent variable, body mass index (normal, overweight, obese and severely obese), and possible covariates, such as age (continuous), gender (male and female), race/ethnicity (Non Hispanic White, Non Hispanic Black, Mexican American, and Others), education level (less than high school, high school/GED, higher than high school), diabetes (yes, no), poverty level (poor, not poor), and marital status (single, married). Means and standard deviations were used to represent continuous variables and proportions were used to represent categorical variables. The Pearson's chi square (categorical) and student's *t* test (continuous) were selected to determine the relationship between two variables. The difference was considered significant when p≤0.05.

Multivariate logistic regression was used to examine the association between the dependent variable HSV infection and independent variables before and after the adjustment for possible confounding factors. The association between variables was represented by odds ratios with 95% confidence interval and *p* values less or equal to 0.05 was considered statistically significant.

### Ethics Statement

The NCHS strictly comply with several laws and regulation addressing participant's confidentiality. Prior to data collection for NHANES, NCHS obtained an approval from NCHS Research Ethics Review Board (formerly the Institute Review Board) [27].

## Results

Table 1 characterizes the study sample. The prevalence of HSV1 infection in U.S. population between 2007 and 2008 was 60.3% (*n* = 1,536). The average age of participants with HSV1 infection was 36.1±0.28. Approximately 62.0% of the females surveyed were HSV1 positive, as were 54.6% of NonHispanic Whites, 63.4% of Non-Hispanic Blacks, 81.1% of Mexican Americans, and 69.0% of individuals who identified themselves as from other racial/ethnic group tested positive for HSV1. Further, 75.3% individuals who had education less than high school, 68.6% who had high school diploma, 52.0% who had higher than High school education, 56.9% of individuals who were single, 62.3% who were married, 68.9% who were classified as poor, 55.6% who were classified above poverty level, 68.9% who reported to be diagnosed with diabetes, and 60.3% who reported not being diagnosed with diabetes also tested positive for HSV1. Age, race/ethnicity, education, marital status and poverty level tested statistically significant (*p*<0.05). Table 2 provides data on the distribution of BMI of the participants by status of HSV1 infection using bivariate analysis. The average BMI in HSV1 positive individuals was 28.6±0.24 and the average BMI in HSV negative individuals was 27.6±0.29. The difference in BMI between HSV1 positive and HSV1 negative groups tested significant (p = 0.009) by Pearson's Chi square test. The BMI group with the highest percentage of seropositives was in the obese group, or those people with a BMI of 30 to 39.9.

**Table 1:** Risk of HSV1 by demographic characteristics and covariates.

		*%* of Sample	*%* HSV1+	*p* values
Total population *n* (%)			1536 (60.3)	
Age Mean (*sd*)*			36.1 (0.28)	0.0011
Age groups				
	20–29 (%)	32.0	50.5	
	30–39 (%)	32.0	66.0	
	40–49 (%)	36.0	64.1	
Gender (*%*)				0.2605
	Male	49.5	58.6	
	Female	50.6	62.0	
Race/Ethnicity (*%*)*				0.0000
	NH White	65.0	54.6	
	NH Black	11.4	63.4	
	Mexican American	11.2	81.1	
	Other	12.4	69.0	
Education (%)*				0.0000
	Less than HS	19.3	75.3	
	HS	23.6	68.6	
	Higher than HS	57.1	52.0	
Marital status (%)*				0.0490
	Single	36.5	56.9	
	Married	63.5	62.3	
PIR (%)*				0.0002
	Poor	35.6	68.9	
	Not poor	64.4	55.6	
Diabetes (%)				0.4294
	Yes	4.2	63.6	
	No	95.8	60.3	

*Tests for the difference among the groups were statistically significant (p≤0.05).

**Table 2:** HSV1 status by BMI.

Variable		% HSV1+	% HSV1−	*p* value
BMI Mean (sd)		28.6 (0.24)	27.6 (0.29)	
BMI (%)*				0.009
	Normal (<25)	55.9	44.1	
	Overweight (25–29.9)	57.9	42.1	
	Obese (30–39.9)	68.7	31.3	
	Severely Obese (>40)	63.4	36.6	

*Tests for the difference among the groups were statistically significant (p≤0.05).

The logistic regression analysis was used to examine the relationship between the main independent variable (BMI) and HSV1 infection before and after controlling for confounding variables (Table 3). The bivariate logistic regression analysis demonstrated that BMI of 30–39.9 was significantly associated with HSV1 infection [unadjusted OR = 1.74 (95% CI 1.20–2.51), *p* = 0.006]. When BMI was used in the full model, the multivariate logistic regression revealed that the BMI for the obese group (BMI of 30–39.9) continued to be significantly associated with HSV1 infection in the U.S. population aged 20–49 years [adjusted OR = 1.50 (95% CI 1.06–2.13), *p* = 0.026] even after the adjustment for the independent variables of age, gender, race/ethnicity, marital status, education, and PIR. The relationship for overweight (BMI of 25–29.9) and for severely obese (BMI of >40) groups was not significant (*p*>0.05).

**Table 3:** Unadjusted and adjusted odds of HSV1 infection by variables.

Variable		Unadjusted OR (95% CI)	Crude p value	Adjusted OR (95%CI)	Adjusted p value
BMI					
	Normal (<25)	1 (Reference)		1 (Reference)	
	Overweight (25–29.9)	1.08 (0.83–1.41)	0.520	0.95 (0.72–1.25)	0.674
	Obese (30–39.9)	1.74 (1.20–2.51)	0.006*	1.50 (1.06–2.13)	0.026*
	Severely Obese (>40)	1.37(1.20–2.51)	0.176	1.07 (0.63–1.82)	0.792
Age		1.32 (1.15–1.52)	0.001*	1.40 (1.20–1.63)	0.000*
Gender (%)					
	Male	1 (Reference)		1 (Reference)	
	Female	1.15 (0.89–1.50)	0.261	1.25 (1.00–1.57)	0.049*
Race/Ethnicity (%)					
	NH White	1 (Reference)		1 (Reference)	
	NH Black	1.44 (1.06–1.97)	0.024*	1.38 (1.03–1.84)	0.031*
	Mexican American	3.57 (2.28–5.59)	0.000*	2.90 (1.92– 4.35)	0.000*
	Other	1.85 (1.24–2.77)	0.005*	1.84 (1.26–2.70)	0.004*
Education (%)					
	Less than HS	2.82 (2.00–3.98)	0.000*	2.07 (1.52–2.81)	0.000*
	HS/GED	2.02 (1.42–2.88)	0.001*	1.95 (1.38–2.76)	0.001*
	Higher than HS	1 (Reference)		1 (Reference)	
Marital Status (%)					
	Married	1 (Reference)		1 (Reference)	
	Single	1.25 (1.00–1.57)	0.049*	1.16 (0.93–1.44)	0.163
PIR (%)					
	Poor	1.77 (1.37– 2.28)	0.000*	1.37 (1.05–1.78)	0.023*
	Not poor	1 (Reference)		1 (Reference)	

*Tests for difference among the groups were statistically significant (p≤0.05).

Additionally, unadjusted and adjusted odds ratios were obtained for covariates (Table 3). Results from the multivariate logistic regression analysis suggest that covariates, such as age [adjusted OR = 1.40 (95%CI 1.20–1.63], female gender [adjusted OR = 1.25 (95%CI 1.00–1.57], race [NH Black adjusted OR = 1.38 (95%CI 1.03–1.84), Mexican American adjusted OR = 2.90 (95%CI 1.92–4.35), Other adjusted OR = 1.84 (95%CI 1.26–2.70)], education[Lower than high school adjusted OR = 2.07 (95%CI 1.52–2.81), High school/GED adjusted OR = 1.95 (95% CI 1.38–2.76)], and being poor [adjusted OR = 1.37 (95%CI 1.05–1.78)] were significantly associated with HSV1 infection (*p*<0.05). The relationship did not test significant for marital status (*p* = 0.16).

## Discussion

This quantitative, cross-sectional study was the first to explore whether obesity plays a role in the distribution of HSV1 infection in the U.S. population using data from a national survey. The results from this study indicate that obesity could play an important role in identifying a high risk population for HSV1 infection as having a BMI in the classification “obese” (BMI 30–39.9) was significantly associated with HSV1 infection before [unadjusted OR = 1.74 (95% CI 1.20–2.51), *p* = 0.006] and after adjusted OR = 1.50 (95% CI 1.06–2.13)], *p* = 0.0026] controlling for socio-demographic factors. This means that obese persons were 1.5 times more likely to be infected with HSV1 when compared to infected persons with normal weight. An odds ratio of 1.5 corresponds to a relationship of medium strength and therefore the odds of being obese are stronger than such other already established risk factors, as age (adjusted OR = 1.40), PIR (adjusted OR = 1.37) and female gender (adjusted OR = 1.25). This finding suggests that a BMI of 30–39.9 could be a significant factor in the susceptibility to HSV and could potentially be used as an indicator for susceptibility to HSV1 infection.

Findings in this study are consistent with findings that suggested a correlation between body fat and HSV1 titer (*p* = 0.038) [21]. This study is also consistent with studies that propose HSV1 as a significant risk factor for metabolic disorders and cardiovascular diseases, in which BMI was a major predictor [7], [29 30], . Since obesity is also a major risk factor for these disorders, it is possible that obesity could be a link between HSV1 and metabolic and cardiovascular disorders.

The association of obesity and HSV1 could possibly be explained by predisposition to the altered production of hormones involved in metabolism in obese people that could lead to a change in immune responses. This theory is consistent with findings from several studies which indicate that a dysfunction in the production of hormones involved in metabolism (e.g., leptin) alter immune system responses [32], [33], [34]. Other studies suggest that obesity may be linked to an increased level of herpes virus entry mediators (HVEM), which could potentially increase viral entry [35]. However, due to data limitations with the data, this theory will need to be evaluated further when hormonal and HVEM levels are available.

The multivariate logistic analysis did not reveal a significant association between severe obesity and HSV1 infection (*p* = 0.79). This finding is not consistent with a dose-response relationship between body fat and HSV1 titer found in different study [21]. However, because there were only 6.6% of persons with severe obesity in the sample, it is possible that the relationship could be detected when using a larger sample size.

The relationship was not found to be significant for overweight group (BMI 25–29.9), which suggests that higher level of obesity may be necessary to show the relationship. This is consistent with findings that HSV1 titer increases with fat mass [21]. Future research could examine contributing factors. For example, the distribution of body fat in the body has been shown to contribute to the effects for metabolic disorders [36]. Therefore, it is possible that such factors could possibly play a role in the association of BMI and the susceptibility to HSV1.

Viral infections have been shown to induce inflammatory responses [5] and adipose tissue was shown to released hormones that have been linked to susceptibility to infection in animals and humans [30], [37]. Therefore, it is plausible that obese people could be more susceptible to HSV1 infections. This study provides some exciting findings, however more studies will be necessary to examine why this association was not seen in overweight individuals and persons who were morbidly obese.

Research reports indicate that genital HSV1 is now being identified more frequently and the HSV1/HSV2 ratio is reversing [3], [13]. Therefore, the awareness and preventive measures against HSV1 transmission will need to be emphasized. Preventive efforts should mainly focus on prenatal screening, development of effective vaccines, and establishing a surveillance program to monitor the effectiveness of implemented preventive measures. These results also suggest that preventive measures should be focused on Mexican Americans. One way to prevent mother-child transmission is to incorporate screening programs into prenatal care. Serological screening for HSV antibodies in the mother and her partner could help to plan for appropriate strategies during the delivery and implementing effective counseling. In addition, surveillance programs will need to be developed to monitor the trends and the effectiveness of preventive measures.

Another preventive strategy that could be implemented should focus on the development of effective vaccines against HSV. Currently, vaccines focusing on minimizing of virus shedding are being evaluated [38]. However, more effective vaccines that would prevent HSV acquisition should be developed. The use of preventive vaccine would be beneficial especially for young population, women and obese individuals since they appear to be most vulnerable to HSV infection.

Preventive measures should continue to focus on modification of behavior and improved personal hygiene to minimize the transmission of HSV. Education about the virus transmission and safe sexual practices were demonstrated to be effective [39], [40]. These measures should be emphasized in younger population and the development of preventive measures should be implemented for the most vulnerable population including persons with weight problems and women.

This study had several limitations. First, the design of this study was cross-sectional; therefore, the study does not provide evidence for a causal relationship. On the other hand, this study used the most recent data, which provides the latest prevalence estimates on both HSV1 infection and obesity in the United States. Second, only adults between 20 and 49 years old were used in this study, therefore, results from this study cannot be extended to younger or older populations. Third, in this study, HSV1 status was determined based on the detection of antibodies in blood samples and serological evaluation was not able to determine whether the infection was acute and where the infection was located in the body. However, the serological test was able to detect positive cases that were symptomatic and could identify people who were not aware of being infected with HSV1 [4]. This serological detection may provide more accurate estimates of infected individuals.

### Conclusion

In this study, individuals who were obese exhibited a higher prevalence of HSV1 infection when compared to individuals with normal weight. Women and individuals with racial/ethnic background, other than non Hispanic White, were at higher risk for HSV1 infections and should be the primary target for interventions.

The prevalence of HSV1 was slightly higher that previously reported. New preventive efforts should mainly focus on prenatal screening, development of effective vaccines, and establishing a surveillance program to monitor the effectiveness of implemented preventive measures. Preventive programs should continue to concentrate on modification of negative behaviors and on the improvement of personal hygiene to minimize the transmission of HSV1.
